# Supplementary material for: Indicators of HSV1 Infection, ECM–Receptor Interaction, and Chromatin Modulation in a Nuclear Family with Schizophrenia
Source: J Pers Med. 2023 Sep 18;13(9):1392. doi: 10.3390/jpm13091392 (PMC10532901; doi:10.3390/jpm13091392)
Supplement: Supplementary file 1 [file jpm-13-01392-s001.zip › Supplementary Table S2.pdf]

Supplementary Table S2. Summary of whole-exome sequencing statistics and variants found in study samples

Sample	Number of mapped reads	% of reads on target	Number of identified variants	Number of silent variants	Number of missense mutations	Number of nonsense mutations
I:1	26,906,693	80.6	584,149	26,237	22,177	147
II:1	27,787,983	82.0	533,463	28,758	22,887	162
II:2	23,391,853	82.7	453,084	28,528	22,904	186
